# Author’s failure to read and follow instructions leads to increased trauma to their manuscripts

**DOI:** 10.12669/pjms.343.15633

**Published:** 2018

**Authors:** Shaukat Ali Jawaid, Masood Jawaid

**Affiliations:** 1 Shaukat Ali Jawaid Chief Editor, Pakistan Journal of Medical Sciences, Karachi - Pakistan; 2 Masood Jawaid Associate Editor, Pakistan Journal of Medical Sciences, Karachi - Pakistan

**Keywords:** Authorship, Peer Review, Journal Publishing, Impact Factor, Predatory journals

## Abstract

Authors under pressure to publish to meet some academic requirement are one of the most dangerous pressure group which the editors of good quality peer review biomedical journals have to face every day. Their failure to read and follow instructions for authors which are published on every journal website, lack of training facilities in research methodology, medical writing, and low computer literacy rate leads to increased trauma to their manuscripts. The authors must realize that from submission to final publication manuscripts go through various stages i.e. internal review, editor’s triage, similarity index check, formatting, external review, revision of the manuscripts in the light of reviewers comments and suggestions. It all takes time and there is no short cut. They must plan at least for six to eight months from the date of submission to acceptance and publication and avoid getting trapped by predatory journals which offer quick publication on payment.

Authors who are under pressure to publish remain the most dangerous pressure group which the editors of biomedical journals have to face every day. Often failure to oblige them as there is no short cut and the whole process takes couple of months and even weeks for initial internal review, most often results in e mails which are not at all palatable to read. Publishing a good quality peer review medical journal with financial and human resource constraints remains a most stressful and frustrating job and the problems are compounded in the developing countries.^1^

Recent developments and revolution in information technology while at one hand has made the job of publishing quite easy but at the same time it has also created lot of problems for the editors. They remain under constant pressure as the authors respond to reviewers comments, resubmit revised manuscripts within days and it offers little breathing time for the editors to clear the pending backlog while the new submissions keep on piling.^2^ Publishing a journal is a team work wherein each and every member has to play its role effectively failing which it adversely affects the whole process.^3^

The authors must understand that it is in their own interest that they must carefully read and follow the instructions for authors of the journal to which they intend to submit their manuscript. Look at some of the published papers on its website and try to religiously follow its format. It is also important to look at what type of manuscripts that particular journal accepts. High rejection rate by any journal does not reflect its high standard because the editors have their own priorities while doing initial screening and triage accepting papers for further processing. They most often go for good quality research, innovative procedures, epidemiology studies, Randomized Control Trials, Systemic Reviews or rare case reports etc., Anything which ensures and attracts greater readership and also has more chances of citations thereby improving the Impact Factor of the journal is most often preferred. At a particular time if a general medical journals gets too many submissions in a particular specialty, it is quite likely that not all of them will be accepted for publication despite the fact that they may be good quality research work.

## Fast Track Processing

Since we follow an author friendly policy, sometime ago just to help the authors, we provided them an opportunity of fast track processing of the manuscripts to those who intend to appear in fellowship examination of CPSP or have a requirement of publication before they can defend their PhD thesis by paying fast track processing fee. However, at times there is a tendency on the part of authors to misuse this facility. They generally think that by just paying fast track processing fee, they will succeed in faster publication which is not the case. We now ask them to justify, give reasons why they wish to get it processed on fast track and prove that they have genuine reasons. Just applying for an academic position or promotion to next higher academic positions are not considered genuine reasons for fast track processing. When the authors insist that they are prepared to pay for that, they are politely told that we do not collect charity. Our prime objective remains to publish good quality research and not make money.

## Emotional appeals and pressure tactics

At times the authors try to influence the editor’s decision through various means. “I will be happy if my papers is accepted and published,” I am from brotherly country and hope for a favorable response for early publication” “I am suffering from cancer and cannot wait as my promotion is due”, are some of the tricks they employ to emotionally blackmail and put pressure. Little do they realize that the editors will also be happy only if the manuscripts are of good quality and it will help improve the quality and standard of their journal? Yet another measure adopted by some authors to put pressure on Editors for acceptance and early publication is by sending fast track processing fee or publication charges at the time of submission. They do it despite clear cut instructions on our website not to arrange fast track processing fee or publication charges unless they are asked to do so. For fast track processing, they must seek permission first and find out whether their manuscript can be processed on fast track or not?

Sometimes authors are not well versed in computer use, cannot submit manuscripts on journal website, fail to upload the supplementary files, and do not know how to do proof readings and make corrections, fail to follow the instructions while revising the manuscripts and all this leads to further delays in the whole process. It is not possible for the editors to teach the authors all these things which they have to learn themselves. Most authors also lack reading habits and it is not possible to write without reading. Above all it is the careless attitude on the part of the authors which they have to avoid during the whole process starting from literature search, conducting the study, preparing and submission of the manuscript which creates lot of problems not only for the peer reviewers and editors but also for the authors themselves which they must overcome and avoid at all costs.

## Role of regulatory Bodies

Since regulatory bodies in many developing countries Pakistan being no exception lack in expertise to help, guide, assist and evaluate medical journals, it has multiplied the problems which the editors have to face. We have time again pointed out that instead of offering financial grants to the journals, institutions like HEC should provide training facilities to the editors to help improve their journals. They can be helped to develop journal websites, setting up peer review system, use of manuscript processing, handling software’s like Open Journal System (OJS) or Editorial Manager etc., provide facilities for generating XML files for submission to PubMed Central or helping journals to get Indexed in important databases but so far it has fallen on deaf ears as those in authority were not prepared to work and dishing out money seems quite easy for them.^4^.^5^ The contribution of regulatory bodies like PM&DC as well as HEC has so far been very disappointing but with the new interim set up in the PM&DC, involvement of a few professional medical editors in framing the Terms of Reference for the recognition and evaluation of medical journals, having some uniform policy by HEC and PM&DC, there are hopes that this communication gap between the regulatory bodies and the medical journal editors which has existed since long, will be bridged and the situation will improve in the coming few months.^6^

## Categorization of manuscripts

Some of the unique problems which we face in Pakistan also include categorization of various manuscripts. Though KAP studies, Survey reports can also be original research it however does not carry the same weight like RCTs which is the highest level of evidence. However, since the regulatory bodies will give credit to only original articles, editors have no other option but to put all of them in the same category to help authors. Ideally, they should be accommodated under different sub-headings i.e. Original article, Systemic Reviews, RCTs etc.,^7^ Faculty members at Agriculture universities attempting clinical studies or statisticians writing up clinical papers without the actual involvement of clinicians in these studies also remains a problem as it often lacks scientific language or medical terms. Statisticians have an important role in conduct and interpretation of study results but they should not think that they are capable of writing papers on clinical subjects without the involvement of clinicians. Incomplete submissions also waste lot of time while impatience of authors who start sending e mails asking for the status of their manuscripts soon after submission is also very irritating and annoying for the editors despite the fact that details of processing of manuscripts are always given in the instructions on journal websites. Authors must also refrain from using language ordering the editors to publish their manuscripts in the coming issue. It is important the medical students are exposed to research from the very beginning, institutions should regularly organize workshops on Research Methodology and Medical Writing for the postgraduates, faculty members. Some institutions have already started this. Medical Education departments should be established in every medical and dental institution and it should facilitate the authors before they make submissions. All this will go a long way to minimize the trauma to their manuscripts.

## Authorship issues

Gift authorship is a menace and we do not encourage this intellectual corruption. That is why we in Pakistan Journal of Medical Sciences just accept up to four authors and in case of more than four, the authors are required to submit a certificate from the Ethics Committee or Institutional Review Board of their institutions confirming that all the listed authors are eligible for authorship as per latest guidelines of International Committee of Medical Journal Editors^8^ Of course there are a few exceptions and the editors take the decisions looking at the study and in case of multicenter studies, the number of authors can be more which is understandable. In some cases if the study is in the field of for example neurosurgery and the listed authors also include obstetricians and gynecologists or cardiologists, it is a dilemma. The editors have to look at it carefully, ask for individual contribution of each listed author. At times it is possible and can be permitted because those from other disciplines who may be more experienced authors, might have helped them in writing, preparing the manuscript with significant intellectual contribution which enables them to be included as author but all this has to be carefully looked at before a decision is taken by the Editors. Hence, it is not right to reject such authors from other disciplines straight away without making any investigations. Authors also have to be careful about the length of manuscript, number of references, inclusion of DOI numbers in references for which they must follow the respective journals format and instructions. Delay in arranging publication charges after the manuscript has been accepted and endeavors to sell authorship, requesting for addition and deletion of authors also disrupts the publication schedule.

## Impact Factor(IF)

Yet another important problems faced by the authors is the regulatory body requirements to publish the manuscripts in Impact Factor journals. At present out of the twelve journals from Pakistan which have got an Impact Factor, only three medical journals enjoy IF which puts them under lot of pressure.^9^ Table-I. Impact Factor, it must be mentioned here is not the only one but one of the criteria to judge the standard of a journal. It has its own drawbacks. Despite lot of criticism and its disadvantages, it will continue to have an impact and this aspect cannot be ignored.^10^ Either the regulatory bodies should waive of this condition or help that more journals published from Pakistan enjoy Impact Factor. There are many ways to help the journals provided those in authority are keen to help. Recently Indian University Grants Commission (IUGC) has removed 4305 dubious sub-standard journals from its approved list. The decision was based on findings of a study published by Bhushan Patwardhan and colleagues. IUGC looked at 30,000 publications used for weighing academic performance. These journals were removed because of poor quality, containing incorrect or insufficient information. 11 The investigators also found that most of the manuscripts published in predatory journals came from India and United States was the second largest contributor to these predatory journals.^11^ Feedback from the workshop participants which the authors have been conducting for the last many years also reveal that even the faculty members what to talk of students and postgraduates lack even basic knowledge. They often face problems in selecting a topic, planning a study or literature search and writing manuscripts.^12^

**Table-I:** Journals from Pakistan with Impact Factor (2017)

Rank	Full Journal Title	Journal Impact Factor
1	PAKISTAN VETERINARY JOURNAL	0.813
2	INTERNATIONAL JOURNAL OF AGRICULTURE AND BIOLOGY	0.746
3	PAKISTAN JOURNAL OF BOTANY	0.69
4	PAKISTAN JOURNAL OF AGRICULTURAL SCIENCES	0.609
5	PAKISTAN JOURNAL OF PHARMACEUTICAL SCIENCES	0.649
**6**	**PAKISTAN JOURNAL OF MEDICAL SCIENCES**	**0.696**
7	INTERNATIONAL JOURNAL OF PHARMACOLOGY	0.753
**8**	**JOURNAL OF THE PAKISTAN MEDICAL ASSOCIATION**	**0.616**
9	PAKISTAN JOURNAL OF ZOOLOGY	0.491
10	JOURNAL OF ANIMAL AND PLANT SCIENCES	0.381
**11**	**JCPSP-Journal of the College of Physicians and Surgeons Pakistan**	**0.372**
12	JOURNAL OF THE CHEMICAL SOCIETY OF PAKISTAN	0.327

## Publication Audit

We have been conducting our own publication audit for the last many years and it has been extremely helpful in knowing our strength as well as weaknesses. It also highlights the various challenges we face and helps us to find some practical, feasible solutions.^13^ While over the years the number of submissions from Pakistan as well as overseas has registered an increase, the overall acceptance rate remains under 20% which is because of our limited financial as well as human resources. The manuscripts we do not accept for further processing does not in any way reflect the poor quality and it must be taken in that spirit. Tables II and III

**Table-II:** Country wise submissions during 2017.

Country	Total
United Arab Emirates	4
Australia	1
Bangladesh	1
Bulgaria	1
China	331
Serbia and Montenegro	3
Cyprus	1
Egypt	7
Ethiopia	1
Falkland Islands (Malvinas)	1
France	1
United Kingdom	14
Indonesia	22
India	15
Iraq	9
Iran	103
Italy	2
Jordan	1
Korea	8
Cayman Islands	1
Kazakhstan	2
Lebanon	1
Sri Lanka	1
Libyan Arab Jamahiriya	1
Macedonia	1
Malaysia	6
New Caledonia	1
Nigeria	4
Nepal	1
**Pakistan**	**453**
Palestinian	3
Romania	5
Saudi Arabia	62
Singapore	2
Thailand	2
Turkey	508
Taiwan	1
United States of America	6
South Africa	1

**Grand Total**	**1588**

**Table-III:** City wise submissions from Pakistan during 2017.

City	Total
Bahawalpur	5
Bannu	1
Dera Ismail Khan	3
Faisalabad	23
Gilgit	4
Gujrat	3
Gujrawala	1
Haripur	3
Hyderabad	10
Islamabad	33
Jamshoro	2
Karachi	149
Kohat	1
Lahore	94
Larkana	1
Mardan	1
Mirpurkhas	1
Multan	18
Muzaffarabad	5
Nawabshah	3
Peshawar	32
Quetta	9
Rahim Yar Khan	8
Rawalpindi	23
Sahiwal	2
Sargodha	8
Sheikhpura	1
Sialkot	6
Swabi	2
Swat	1

**Grand Total**	**453**

There has been a slight decrease in the number of submissions during the last year simply because we have now given an option to the authors that if they wish, they can get an opinion on their manuscripts whether they will be acceptable for further processing or not by just sending us an abstract instead of going for complete submissions on journal website. It also saves them from paying the processing fee. Hence, a large number of authors avail this opportunity to get feedback and quick review, hence the decrease in total number of submissions. Figure-I. It also helps us in keeping the inventory minimum and we can concentrate on manuscripts accepted for further processing and external review.

**Fig.1:**
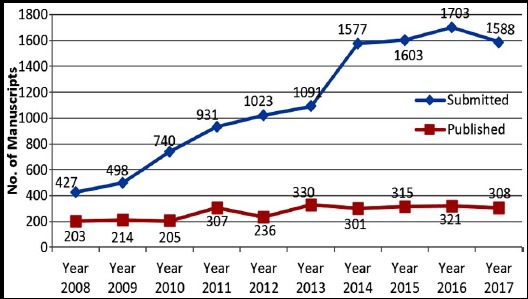
Submissions and acceptance Pakistan Journal of Medical Sciences (2008-2017)

Statistics for the year 2017 also reveal that as usual large number of submissions from overseas came from Turkey, China, Iran, and Saudi Arabia while most submissions from Pakistan came from major cities like Karachi, Lahore, Rawalpindi-Islamabad and Peshawar as usual. Table I& II Total submissions during the year were 1588, number of manuscripts published during the same period were 308, three were rejected because of plagiarism and seventeen were withdrawn by the authors. Table-IV Since we check only those manuscripts for plagiarism using iThenticate software which are accepted for further processing and external review, a few are rejected due to plagiarism. Looking at the similarity index score and plagiarism report in detail, if some manuscripts have slightly increased similarity index score, the authors are advised to revise it before further processing. Details of category wise manuscripts published during the Year 2017 are given in Table-V while details of countries from which the manuscripts were published during the same period are given in Table-VI.

**Table-IV:** PJMS manuscripts statistics of 2017 at a Glance.

2016 Published Articles in 2017:	105
Total Published Articles:	308(19.4%)
Total Rejected Articles:	1233(77.9%)
Total Rejected article on plagiarism:	3
Total Withdraw Articles:	17
Under Process:	132
Total Received Articles:	1588

**Table-V:** Category Wise Manuscript Published in 2017.

Category	Jan-Feb 2017	Mar-Apr 2017	May-Jun 2017	Jul-Aug 2017	Sep-Oct 2017	Nov-Dec 2017	Total
Original Articles	45	43	46	46	41	40	261
Case Reports	2	1	6	1	3	2	15
Correspondence/Letter	2		1				3
Clinical Case Series	1	2	1	1		1	6
Guest Editorial	1		1			1	3
Brief Comm.		1					1
Editorial		1			1		2
Review Article		1		2	1		4
Short Comm.		1	1		1	4	7
Special Comm.		1			1		2
View Point		1					1
Publication Audit		1					1
Systematic Review				2			2

**Grand Total**	**51**	**53**	**56**	**52**	**48**	**48**	**308**

**Table-VI:** Country Wise Manuscript Published in 2017.

Country	Jan-Feb 2017	Mar-Apr 2017	May-Jun 2017	Jul-Aug 2017	Sep-Oct 2017	Nov-Dec 2017	Total
Pakistan	25	24	27	28	29	30	162
China	9	8	11	12	5	9	54
Turkey	9	10	8	8	7	7	49
Saudi Arabia	4	4	6	1	5	1	21
Iran	2	3	2	1	2		10
United Kingdom	1						1
Romania	1						1
Korea		1	1				2
Malaysia				1			1
USA		1	1				2
United Arab Emirates				1			1
Australia		1					1
Japan		1					1
Serbia & Montenegro						1	1

**Grand Total**	**51**	**53**	**56**	**52**	**48**	**48**	**308**

Though we need to promote research culture by providing all the help and facilities to the young researcher’s, medical institutions but at the same time it won’t be fair to put the postgraduates and faculty members under too much stress. The emphasis should be on quality of research and not quantity of papers which has unfortunately also given birth to lot of predatory journals. Publish or Perish needs to be given another look by the authority’s concerned.^14^ At present many impatient authors under pressure to publish get trapped by predatory journals offering quick publication on payment. These predatory journals make all sort of dubious claims of having high Impact Factor using the terms like Global Impact Factor, Scientific Impact Factor or Universal Impact Factor etc., and authors need to be extremely careful and do not get trapped. The whole publication process starting from submission to internal review, editor’s triage, similarity index check, formatting, external review, sharing the reviewer’s comments with the authors, revision of manuscripts by the authors responding to the reviewer’s comments and suggestions is a lengthy long drawn procedure and there is no short cut. However, it offers numerous advantages. While on one hand it is an effective barrier against the spread of wrong information, it also helps improve the quality of manuscripts before they are published.

## References

[1] Jawaid SA (2004). Problems faced by editors of peer reviewed medical journals. Saudi Med J.

[2] Jawaid SA, Jawaid M (2013). Are the Editors faced with e-problems performing their duties and responsibilities satisfactorily?. Pak J Med Sci.

[3] Jawaid SA, Jawaid MA (2017). How to run a successful Journal. Pak J Med Sci.

[4] Jawaid SA (2016). Professionalism in Medical Journalism and Role of HEC, PM&DC. Annals of King Ed Med Univ.

[5] Jawaid SA, Jawaid M (2017). What regulatory agencies like HEC, PM&DC can do to help improve quality and standard of Pakistani Biomedical Journals. Pak J Med Sci.

[6] Pledges to restore the Glory of PM&DC as a regulatory body Pulse International May 1, 2018.

[7] Jawaid SA (2017). Importance of Publication Audit, fast track processing and categorization of manuscripts. Pak J Med Sci.

[8] Defining the Role of Authors and Contributors.

[9] Journal Data Filtered By:Selected JCR Year:2016 Selected Editions:SCIE, SSCI Selected Countries:'PAKISTAN'Selected Category Scheme:WoS. Clarivate Analytics.

[10] Jawaid SA (2014). Despite misuse and abuse, Journal Impact Factor will retain its impact and won't fade away soon. (Editorial) JPMI Peshawar.

[11] India culls 4305 Dubious Journals from approved list.

[12] Jawaid M, Zubia M, Alam SN, Jawaid SA (2011). An analysis of interactive hands-on workshops on medical writing. J Pak Med Assoc.

[13] Jawaid SA (2015). Publication Audit - a useful tool to evaluate progress and plan for the future. Pak J Med Sci.

[14] Jawaid SA (2016). Publish or Perish:Need to have another look?. Pak J Med Sci.

